# Esterase-Mediated Pyrethroid Resistance in Populations of an Invasive Malaria Vector *Anopheles stephensi* from Ethiopia

**DOI:** 10.3390/genes15121603

**Published:** 2024-12-15

**Authors:** Daibin Zhong, Teshome Degefa, Guofa Zhou, Ming-Chieh Lee, Chloe Wang, Jiale Chen, Delenasaw Yewhalaw, Guiyun Yan

**Affiliations:** 1 Joe C. Wen School of Population & Public Health, University of California at Irvine, Irvine, CA 92697, USA; zhoug@uci.edu (G.Z.); mingchil@uci.edu (M.-C.L.); xiaomiw1@hs.uci.edu (C.W.); jialechen873@ucsb.edu (J.C.); guiyuny@uci.edu (G.Y.); 2 School of Medical Laboratory Sciences, Institute of Health, Jimma University, Jimma MVJ4+R95, Ethiopia; teshedege@gmail.com (T.D.); delenasawye@yahoo.com (D.Y.); 3 Tropical and Infectious Diseases Research Centre (TIDRC), Jimma University, Jimma MVJ4+R95, Ethiopia

**Keywords:** *Anopheles stephensi*, invasive malaria vector, RNA-seq, differentially expressed genes, co-expression network, Ethiopia

## Abstract

Background: The swift expansion of the invasive malaria vector *Anopheles stephensi* throughout Africa presents a major challenge to malaria control initiatives. Unlike the native African vectors, *An. stephensi* thrives in urban settings and has developed resistance to multiple classes of insecticides, including pyrethroids, organophosphates, and carbamates. Methods: Insecticide susceptibility tests were performed on field-collected *An. stephensi* mosquitoes from Awash Sebac Kilo, Ethiopia, to assess insecticide resistance levels. Illumina RNA-seq analysis was then employed to compare the transcriptomes of field-resistant populations and susceptible laboratory strains (STE2). Results: *An. stephensi* populations exhibited high levels of resistance to both deltamethrin (mortality, 39.4 ± 6.0%) and permethrin (mortality, 59.3 ± 26.3%) in WHO tube bioassays. RNA-seq analysis revealed that both field-resistant and field-unexposed populations exhibited increased expressions of genes associated with pyrethroid resistance, including esterases, P450s, and GSTs, compared to the susceptible STE2 strain. Notably, esterase E4 and venom carboxylesterase-6 were significantly overexpressed, up to 70-fold, compared to the laboratory strain. Functional enrichment analysis revealed a significant overrepresentation of genes associated with catalytic activity under molecular functions and metabolic process under biological process. Using weighted gene co-expression network analysis (WGCNA), we identified two co-expression modules (green and blue) that included 48 genes strongly linked to pyrethroid insecticide resistance. A co-expression network was subsequently built based on the weight values within these modules. Conclusions: This study highlights the role of esterases in the pyrethroid resistance of an *An. stephensi* population. The identification of candidate genes associated with insecticide resistance will facilitate the development of rapid diagnostic tools to monitor resistance trends.

## 1. Introduction

The rapid spread of the invasive malaria vector, *An. stephensi*, across Africa poses a significant threat to malaria control efforts. Originating from Southeast Asia and the Arabian Peninsula, this mosquito species has rapidly colonized multiple African countries such as Djibouti, Ethiopia, Somalia, Kenya, Sudan, Nigeria, and Ghana, leading to an increase in malaria cases [1,2]. Unlike the native African vectors, *An. stephensi* thrives in urban settings and has developed resistance to multiple classes of insecticides, including pyrethroids, organophosphates, and carbamates [3,4,5]. This widespread resistance undermines the effectiveness of the traditional vector control measures, such as long-lasting insecticidal nets (LLINs) and indoor residual spraying (IRS) [6,7]. To effectively manage insecticide resistance, it is crucial to monitor resistance patterns and understand the underlying mechanisms.

Insecticide resistance is primarily driven by two mechanisms: target-site insensitivity and metabolic resistance [8]. Target-site insensitivity, particularly mutations in the voltage-gated sodium channel (*VGSC*) gene, confers resistance (knockdown resistance, *kdr*) to pyrethroids and DDT. Mutations and duplications in the acetylcholinesterase 1 (*Ace-1*) gene have been associated with resistance to carbamates and organophosphates, while mutations in the GABA receptor (*Rdl*) gene confer resistance to phenylpyrazoles and organochlorines. Additionally, metabolic resistance, which is often associated with the overexpression of detoxification enzymes, can contribute to resistance to multiple insecticide classes. Other potential mechanisms, such as alterations in the mosquito microbiome and cuticle, may also play a role [9,10].

In *An. stephensi*, several target-site mutations associated with insecticide resistance have been detected. These include the *kdr*-West (L1014F) and *kdr*-east (L1014S) mutations in the voltage-gated sodium channel gene, the N177D mutation in the acetylcholinesterase 1 (*Ace-1*) gene, and the A296S mutation in the GABA receptor (*Rdl*) gene [11,12,13,14]. However, the frequency of these mutations is generally low. For instance, the *kdr*-West mutation has been reported at low frequencies in various populations of different countries [11,14,15,16]. Additionally, the V189L mutation in the *GSTe2* gene has been detected at a low frequency in *An. stephensi* populations from Ethiopia [13]. These findings suggest that while target-site mutations contribute to insecticide resistance in *An. stephensi*, other mechanisms, such as metabolic resistance, may play a more significant role. Increased activity of detoxification enzymes, including glutathione S-transferases (GSTs), esterases, and cytochrome P450s, has been implicated in insecticide resistance [17]. Additionally, alterations in membrane transporters, such as ABC transporters, may contribute to reduced insecticide penetration [18,19]. Sanil et al. (2014) demonstrated a strong association between increased GST activity and resistance to multiple insecticide classes, including pyrethroids, organophosphates, organochlorines, carbamates, and biocides [20]. Recently, RNA-seq technology has been utilized to identify genes that are differentially expressed in *An. stephensi* larvae following permethrin exposure [21]. However, comprehensive whole-transcriptome analyses specifically focusing on the molecular mechanisms of pyrethroid resistance in the newly invasive *An. stephensi* population have not yet been conducted.

In this study, we performed standard World Health Organization (WHO) bioassays to evaluate the insecticide susceptibility of a newly invasive field population of *An. stephensi* from Ethiopia. To investigate the molecular mechanisms underlying insecticide resistance, we conducted a whole-transcriptome analysis comparing the gene expression profiles of field-resistant populations with a susceptible laboratory strain (STE2). We further compared the transcriptomic profiles of field-unexposed populations to identify shared patterns of gene expression associated with insecticide resistance.

## 2. Materials and Methods

### 2.1. Mosquito Larval Sampling and Insecticide Susceptibility Bioassay

*Anopheles* mosquitoes were sampled from Awash Subac Kilo, a market town located in central Ethiopia at an altitude of approximately 1000 m. With a population of 34,669 (as of July 2021), this region experiences a hot, semi-arid climate characterized by minimal rainfall and a short wet season from July to September. Both *Plasmodium falciparum* and *Plasmodium vivax* malaria parasites are present in this region. In June–July 2022, *Anopheles* larvae and pupae were collected from diverse larval breeding habitats, including concrete water cisterns, water tanks, steel drums, plastic containers, plastic sheet water storage at construction sites, and discarded buckets. To minimize the collection of related individuals, no more than ten specimens were collected from each habitat using a standard 350 mL-capacity mosquito dipper. The collected larvae and pupae were pooled and reared to adulthood in an insectary for subsequent bioassays. Prior to the insecticide susceptibility bioassay, the morphological identification of *Anopheles* individuals was performed while the mosquitoes were alive (with a brief chill on ice if needed) following the taxonomic keys [22]. Adult female *An. stephensi* mosquitoes (3–5 days old) were then used in the bioassay, where they were exposed to pyrethroid insecticide-impregnated papers (0.05% deltamethrin and 0.75% permethrin) at diagnostic doses using WHO test tubes in a bio-secure insectary. Each insecticide was tested with batches of 25 female *An. stephensi* mosquitoes in both test and control tubes. At least 100 mosquitoes were exposed to each insecticide in four replicates (25 per replicate) for 1 h. Mortality was assessed 24 h after exposure. Abbot’s formula was applied to adjust for control mortality when needed. According to the WHO guidelines, mosquito populations were classified as “resistant” if the mortality rates were below 90%, as “suspected resistant” if the mortality rates were between 90% and 98%, and “susceptible” if the mortality rates were > 98% [8]. The *An. stephensi* mosquitoes that survived the insecticide exposure test, along with unexposed samples, were promptly killed by placing them in a deep freezer for about 30 min until complete knockdown occurred. The samples were immediately stored in 0.5 mL Eppendorf tubes with RNALater for subsequent molecular and whole transcriptome analysis.

### 2.2. Molecular Identification of Anopheles Mosquito Species

DNA was extracted from a single leg of a subset of *Anopheles* mosquitoes to confirm species via quantitative polymerase chain reaction (qPCR). The highly sensitive TaqMan probe-based qPCR method was used to identify *An. arabiensis* and *An. stephensi* [23,24]. A total of 480 mosquitoes were tested for molecular confirmation of species identification. Additionally, a subset of 20 mosquitoes from each species underwent Sanger sequencing of ITS2 PCR products to further validate species identity [25].

### 2.3. RNA Extraction and Transcriptome Sequencing (RNA-seq)

RNA was extracted individually from female *An. stephensi* samples using column-based RNA extraction kits (cat # 2050, Zymo Research, Irvine, CA, USA). The RNA was assessed for quality using agarose gel electrophoresis (1%) and quantified with the Qubit 3.0 RNA HS Assay (ThermoFisher, Waltham, MA, USA). The RNA samples comprised three groups: field deltamethrin-resistant (AWR) mosquitoes, field-unexposed control (AWK) mosquitoes, and a susceptible laboratory strain (STE2: BEI Resources, Cat# MRA-128). For each group, 10 RNA samples were pooled in equal amounts to create 1 composite sample. Specifically, 200 ng of RNA from each sample was combined, and the final volume was adjusted to 100 µL per pool with nuclease-free water, resulting in a pooled RNA concentration of 200 ng/µL. Two replicates/pools were prepared per group. In total, 6 pools comprising 60 mosquitoes were generated for RNA library preparation and RNA-seq. The IDT xGen RNA library prep kit (Coralville, IA, USA) with Illumina Ribo-zero Plus (San Diego, CA, USA) was used for library preparation. Final quality control was performed using Kapa qPCR (Roche, Basel, Switzerland) and Agilent TapeStation (Santa Clara, CA, USA). The libraries were sequenced on an Illumina^®^ NovaSeq S4-PE150 (San Diego, CA, USA).

### 2.4. Data Filtering, Mapping Reads, and Transcriptome Analysis

The generated sequences underwent an initial filtering process. Raw paired-end sequence reads were assessed for quality using FASTQC (v0.12.1) [26], and Trimmomatic (v.0.39) [27] was employed to remove Illumina adapters. After quality control and filtering, the sequence reads were analyzed using CLC Genomics Workbench v23 (CLC Bio, Cambridge, MD, USA), with the reference genome VectorBase-68_AstephensiUCISS2018. RNA-seq analysis was conducted using the RNA-seq tool and the reference genome annotated with genes and transcripts, applying the following parameters: mismatch cost = 2, insertion cost = 3, deletion cost = 3, length fraction = 0.8, similarity fraction = 0.8, maximum number of hits per read = 10, count paired reads as two = no, ignore broken pairs = yes, and expression value = RPKM. The Differential Expression in Two Groups tool in CLC was used to conduct a statistical differential expression analysis of a set of expression tracks and a control. The experiments included two biological replicates for both the field population group and the lab strain control group. The raw gene expression matrix was transformed into a normalized gene expression matrix. The biological coefficient of variation (BCV), representing biological variability, was estimated using edgeR, which models RNA-seq data variability through a negative binomial distribution [28]. Differentially expressed genes (DEGs) were identified using the Differential Expression in Two Groups tool in CLC, with the STE2 group used as the control. All RNA-seq data were screened for the false discovery rate (FDR), and the results were deemed valid if the FDR was less than 0.05 [29]. DEGs were determined based on a |fold-change| > 1.5 and false discovery rate (FDR) < 0.05. The ShinyGo bioinformatics tool was used for functional annotation and pathway analysis of the DEGs [30].

### 2.5. Gene Co-Expression Network Construction with WGCNA

Gene co-expression network analysis was conducted using the WGCNA package (version 1.72) in R based on differentially expressed genes (DEGs) [31]. The soft threshold (power) was determined using the “pickSoftThreshold” function to generate a scale-free network for both population datasets. The cluster dendrogram and co-expression gene module tree for DEGs were created using the “Dynamic Tree Cut” and “Merged Dynamic” methods. The module eigengene (ME), representing the expression profiles of the module genes, was calculated using WGCNA. Gene co-expression networks were then independently constructed with the "blockwise Modules" function using these parameters: power = 28, minModuleSize = 30, deepSplit = 2, and mergeCutHeight = 0.3. The edge and node data of the target module were exported with the “export Network To Cytoscape” function to build a co-expression network, which was visualized using Cytoscape software (Version 3.10.2) [32].

## 3. Results

### 3.1. Species Composition and Pyrethroid Susceptibility of An. stephensi

Molecular identification of the *Anopheles* species indicated that 88.3% were *An. stephensi* and 11.7% were *An. arabiensis* (*n* = 480), with no other *Anopheles* species identified. High levels of pyrethroid resistance were detected in the *An. stephensi* field population, with mortality rates falling below 60% for both tested insecticides. The mortality rate for permethrin was 59.3% ± 26.3% (mean ± SD, *n* = 8), while for deltamethrin, it was 39.4% ± 18.9% (mean ± SD, *n* = 10) (Figure S1). The estimated coefficient of variation (CV%) among the biological replicates was 47.9% for permethrin and 44.3% for deltamethrin, respectively.

### 3.2. RNA Sequencing and Preliminary Analysis of the Raw Data

RNA-seq was performed using three different groups: field-resistant (AWR), field-unexposed (AWK), and lab strain (STE2), with two independent biological replicates per group. The RNA used for library construction had to pass quantity and quality control criteria. Six libraries were constructed from the three different groups, and raw reads were produced from the six libraries using the Illumina sequencing platform. The RNA-seq generated a total of 70–85 million reads per sample, with an average of 80.6 ± 23.6 million reads. After trimming, 57.5% ± 0.7% of paired end reads, 19.5% ± 0.2% of forward reads, and 9.4% ± 0.3% of reverse reads were retained, while 14.1% ± 0.5% of reads were discarded (Table 1).

### 3.3. Differentially Expressed Gene Analyses

**Figure 1:**
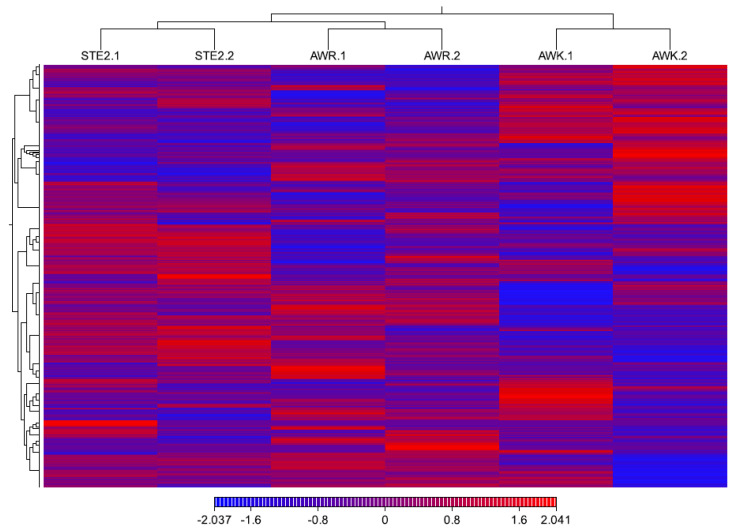
Heatmap of gene expression patterns for each population.

**Figure 2:**
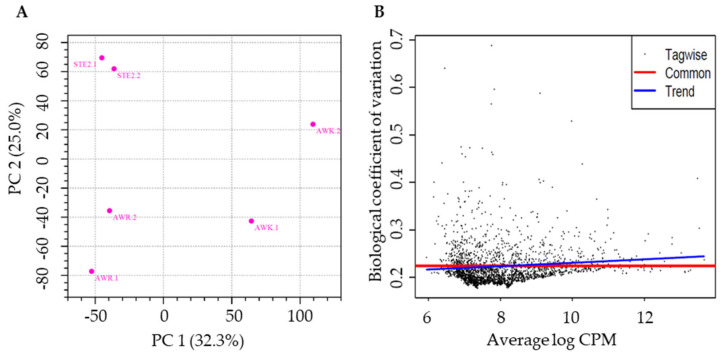
Principal component analysis (PCA) and biological coefficient of variation (BCV) plot of RNA-seq data. (**A**) PCA of gene expression dataset; (**B**) gene expression displayed against the average log CPM (counts per million), as estimated by edgeR. The blue line represents the global or abundance-dependent dispersion trend, while the red line indicates the common dispersion value.

The cleaned, high-quality RNA-seq reads were used to evaluate the differences in gene expression (Figure 1), with the FPKM values calculated to quantify the expression levels across all genes in the three groups (six pooled samples). The raw gene expression matrix was transformed into a normalized gene expression matrix (Figure S2). A total of 10,568 protein-coding genes (83.2% of the 12,705 total genes) were expressed at a minimum exon read count of five across the six pooled samples (Table S1). Principal component analysis (PCA) of the RNA-seq data revealed greater variation among the groups than within the groups (Figure 2A). The estimated coefficient of variation was 0.0517, corresponding to a biological coefficient of variation (BCV) of 0.2273. This BCV value is lower than that typically observed in other studies for well-controlled experiments (BCV = 0.4) (Figure 2B) [33], indicating the stability and reliability of the RNA-seq data.

**Figure 3:**
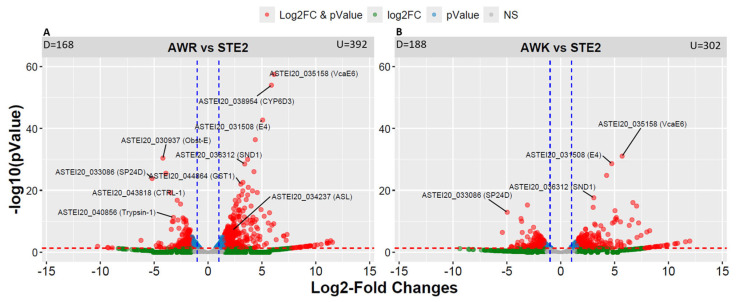
Volcano plot showing the transcripts identified by RNA-seq analysis of an *An. stephensi* population. (**A**) AWR vs. STE2; (**B**) AWK vs. STE2. The gray dots represent the genes that do not meet the criteria for log2 fold change (FC) > 1.5 (up or down) or a significant adjusted *p* value < 0.05. The green dots represent the genes that meet the criterion for log2 FC > 1.5 (up or down) but not an adjusted *p* value < 0.05. The blue dots represent the genes with an adjusted *p* value < 0.05 but not log2 FC > 1.5 (up or down). The horizontal red dashed line is located at a value equivalent to the adjusted *p* value (0.05). The vertical blue dashed lines are located at + and − 1.5 log2 FC. Several highly differentially expressed genes across geographic locations are also labelled. D, down-regulated; U, up-regulated.

Differential gene expression analysis identified 885 significantly differentially expressed genes (DEGs) between the two groups (Table S2). Specifically, 560 DEGs were found between AWR and STE2, with 168 downregulated and 392 upregulated genes (Figure 3A). Similarly, 490 DEGs were identified between AWK and STE2 (Figure 3B). These results provide insights into the molecular mechanisms underlying the phenotypic differences between the two groups.

### 3.4. Classification of the Differentially Expressed Genes

Cluster analysis of the 885 differentially expressed genes revealed distinct patterns of gene expression between the two groups (Figure S3). Approximately one-third of the genes (165) were shared between the two groups, while the remaining two-thirds (395 and 325) were unique to each group, indicating significant genetic divergence among the populations (Figure S4).

A total of 20 genes involved in metabolic detoxification (including P450s, GSTs, esterases, UGTs, and others) were identified and consistently differentially expressed (either upregulated or downregulated) across field-resistant, field-unexposed, and susceptible laboratory *An. stephensi* populations (Table 2 and Table S2). Notably, genes from the esterase family, including venom carboxylesterase-6 and esterase E4, exhibited the most significant upregulation (up to 70-fold) in the resistant populations compared to the susceptible strain. Additionally, four P450 genes and three UGT genes were consistently upregulated. In contrast, a few genes, such as chorion peroxidase and protein arginine N-methyltransferase 5, were downregulated (Table 2).

### 3.5. Functional Annotation of the Differentially Expressed Genes

**Figure 4:**
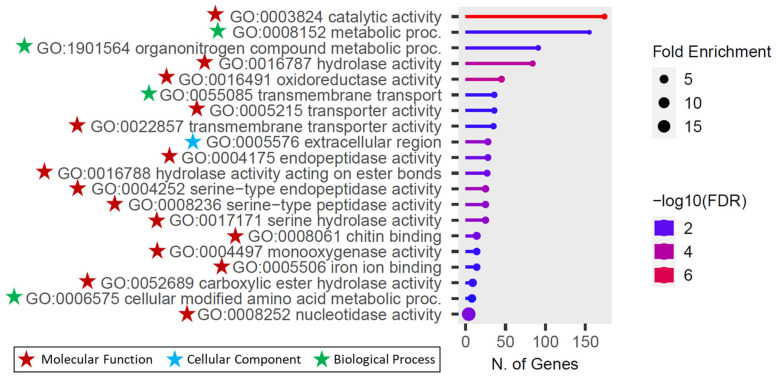
Gene Ontology (GO) term enrichment analysis of genes differentially expressed between pyrethroid resistant mosquitoes and susceptible lab strain (STE2). Significantly enriched GO terms were selected based on an FDR cutoff of 0.05.

**Figure 5:**
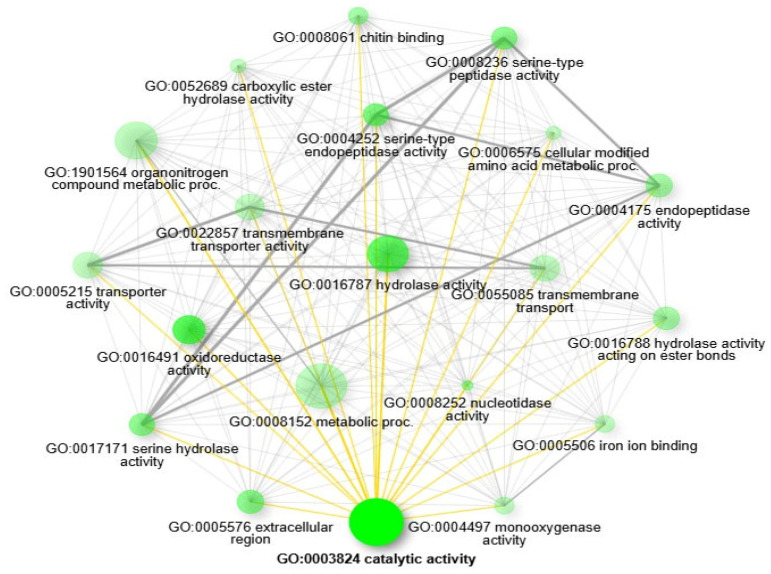
The relationship between enriched pathways. Darker nodes are more significantly enriched gene sets. Bigger nodes represent larger gene sets. Thicker edges represent more overlapped genes.

Gene Ontology (GO) enrichment analysis was conducted to functionally annotate the differentially expressed genes (DEGs) based on the *An. stephensi* SDA-500 annotation. The GO analysis identified a total of 27 enriched GO categories, which included 9 biological processes, 1 cellular component, and 17 molecular functions (Table S3). The most significantly enriched categories were in the catalytic activity under molecular functions and metabolic process under biological process (Figure 4). Furthermore, the pathway network analysis of functional linkages highlighted the top 20 GO enrichments, including GO:0003824 catalytic activity, GO:0016491 oxidoreductase activity, GO:0016787 hydrolase activity, GO:0004252 serine-type endopeptidase activity, GO:0008236 serine-type peptidase activity, GO:0017171 serine hydrolase activity, GO:0005576 extracellular region, GO:0008252 nucleotidase activity, GO:0008061 chitin binding, GO:0004175 endopeptidase activity, GO:0016788 hydrolase activity acting on ester bonds, GO:1901564 organonitrogen compound metabolic proc., GO:0008152 metabolic process, GO:0052689 carboxylic ester hydrolase activity, GO:0004497 monooxygenase activity, GO:0055085 transmembrane transport, GO:0005506 iron ion binding, GO:0022857 transmembrane transporter activity, GO:0005215 transporter activity, and GO:0006575 cellular modified amino acid metabolic proc (Figure 5).

### 3.6. Construction of Gene Co-Expression Network

**Figure 6:**
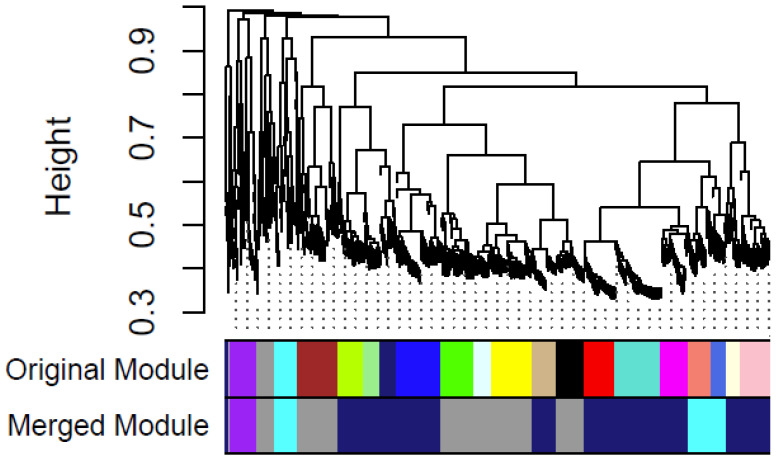
Gene co-expression module identification using the dynamic tree cut method.

**Figure 7:**
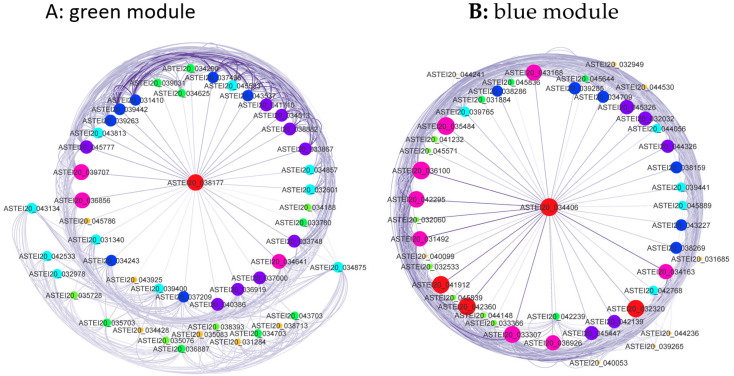
Co-expression network of the genes related to pyrethroid insecticide resistance in *An. stephensi*. Node size and coloring depth represent node connectivity. The coloring line depth represents the weight value. The red dots indicate highly interacting gene clusters. (**A**) Green module; (**B**) blue module.

A soft threshold (power) of 28 was applied to construct a scale-free network for the differentially expressed genes (Figure S5A,B). From the 560 differentially expressed genes between the field-resistant population and the susceptible lab strain, 5 gene co-expression modules were identified from the original 20 modules (Figure 6). The blue and green modules exhibited consistent expression patterns across replicates (Figure S6). Most genes in the green module were upregulated, while those in the blue module were downregulated. A total of 48 genes in each module showed co-expression (Tables S4 and S5). Figure 7 presents the co-expression network of genes associated with pyrethroid insecticide resistance in *An. stephensi*, with edge weight thresholds of 0.40 and 0.68, respectively.

## 4. Discussion

The recent invasion of *An. stephensi* in Africa poses a significant threat to malaria control efforts. To effectively combat this invasive vector, it is crucial to assess its susceptibility to insecticides and understand the mechanisms underlying its resistance. This study aimed to investigate the molecular basis of insecticide resistance in *An. stephensi* populations from Ethiopia. Through RNA-seq analysis, we identified significant upregulation of esterase E4 and venom carboxylesterase-6 in the field-resistant populations compared to a susceptible laboratory strain. While the *kdr* mutation was reported at low frequencies, our findings suggest that metabolic resistance, mediated by detoxification enzymes, could be a primary driver of insecticide resistance in *An. stephensi*. These insights emphasize the need for implementing insecticide resistance management strategies, which include integrated vector management to combat this growing challenge.

Esterases are enzymes that catalyze the hydrolysis of ester bonds. Esterase E4 has been previously implicated in insecticide resistance in other insects [34]. Recent studies have highlighted the potential role of venom carboxylesterase-6-like genes in insecticide resistance. For instance, Wang et al. (2023) identified a potential insecticide resistance marker within this gene in Bemisia tabaci [35]. Additionally, Kang et al. (2021) identified venom carboxylesterase-6-like as a critical odorant-degrading enzyme (ODE) in *Aphidius gifuensis* [36]. In our study, we observed significantly higher expression of venom carboxylesterase-6-like (a 52- to 70-fold increase) and esterase E4 (a 26- to 33-fold increase) in the field population. This suggests that these genes may play a role in the detoxification of pyrethroid insecticides. Additionally, several other metabolic detoxification genes were found to be overexpressed, including monooxygenases (e.g., *CYP6D3*), glutathione S-transferases (e.g., *GST1*), and immunity genes (e.g., *scaf*, *SP24D*, *SND1*).

Next-generation sequencing (NGS), particularly RNA-seq, has revolutionized transcriptomic analysis by providing a comprehensive and unbiased view of gene expression [37,38]. Despite advancements, challenges persist, especially with organisms that have high rRNA content, such as archaea and certain insects [39,40,41]. Although RNA-seq protocols have greatly improved, residual rRNA contamination can still affect data quality, particularly in studies using kits developed for mammalian samples, where rRNA removal may be less specific in non-target organisms like mosquitoes. In our study, we successfully employed RNA-seq to identify differentially expressed genes in *An. stephensi* mosquitoes despite these limitations. While the Illumina Ribo-Zero Plus rRNA Depletion Kit proved effective, residual rRNA contamination was observed, highlighting the need for optimized protocols for non-standard organisms.

This study demonstrated that pooled DNA-seq is a cost-effective and powerful tool for analyzing RNA-seq data in field populations of *An. stephensi*. Pooling ten mosquito samples helped to reduce technical variability among individual samples. However, this approach has limitations, including reduced statistical power due to fewer effective replicates and potential challenges in detecting low-abundance transcripts and rare variants [42]. Additionally, inaccuracies in sample pooling, such as uneven RNA quantities, can introduce bias into the analysis [43]. To mitigate these limitations, careful experimental design, rigorous data analysis, and appropriate statistical methods are essential.

In Ethiopia, *An. stephensi* populations have developed resistance to various insecticides. Several target-site mutations, including *kdr*-West (L1014F) in the voltage-gated sodium channel, N177D in acetylcholinesterase 1 (*Ace-1*), and A296S in the *GABA* receptor (*Rdl*), have been detected in these populations [11,12,13]. While our RNA-seq analysis provides valuable insights into gene expression patterns, it was not optimal for SNP detection due to the limitations of pooled RNA-seq, such as reduced genomic coverage and potential allele frequency bias. To address these limitations, future studies should focus on high-quality RNA extraction and increased sequencing depth to enable accurate SNP detection and analysis.

## 5. Conclusions

The rapid spread of the invasive malaria vector *An. stephensi* poses a significant threat to malaria control efforts in Africa. The emergence of insecticide resistance in this species further complicates vector control strategies. Our RNA-seq analysis revealed that *An. stephensi* populations from Ethiopia exhibited elevated expression of genes associated with pyrethroid resistance, including esterases (e.g., esterase E4 and venom carboxylesterase-6), P450s, and GSTs. These findings suggest that metabolic resistance plays a crucial role in the development of insecticide resistance in this species. Functional validation of key genes involved in insecticide resistance, coupled with the development of improved resistance markers, will be essential for monitoring resistance trends and guiding future control efforts.

## Figures and Tables

**Table 1:** RNA-seq read filtering statistics for *An. stephensi* field population from Ethiopia and susceptible lab strain.

Sample	Population	Input Raw Reads (PE150)	Both Surviving	%	Only Forward Surviving	%	Only Reverse Surviving	%	Dropped	%
AWK.1	Unexposed	84,895,174	47,073,550	55.5%	16,517,385	19.5%	8,087,876	9.5%	13216363	15.6%
AWK.2	Unexposed	70,500,535	40,077,550	56.9%	13,039,840	18.5%	7,395,075	10.5%	9,988,070	14.2%
AWR.1	Resistant	77,387,200	44,172,164	57.1%	14,860,161	19.2%	7,087,466	9.2%	11,267,409	14.6%
AWR.2	Resistant	81,621,753	49,604,580	60.8%	15,114,460	18.5%	6,958,266	8.5%	9,944,447	12.2%
STE2.1	STE2 (Lab)	83,335,260	46,749,249	56.1%	15,935,401	19.1%	8,002,670	9.6%	1,2647,940	15.2%
STE2.2	STE2 (Lab)	85,893,152	50,424,191	58.7%	16,746,130	19.5%	7,601,598	8.9%	11,121,233	13.0%
Average		80,605,512	46,350,214	57.5%	15,368,896	19.1%	7,522,159	9.4%	11,364,244	14.1%
Standard Error (SE)		2,361,586	1,548,772	0.8%	556,320	0.2%	189,764	0.3%	549,434	0.5%

**Table 2:** List of the 20 consistently expressed detoxification enzyme genes in field-resistant and field-unexposed *Anopheles stephensi* populations.

Gene ID	Gene Description	Length (bp)	R vs. S (FC)	U vs. S (FC)	Gene Expression
ASTEI20_031161	probable cytochrome P450 6a14	1797	3.62	3.60	up
ASTEI20_031508	esterase E4, transcript variant X2	2198	33.67	26.56	up
ASTEI20_031614	probable cytochrome P450 9f2	2220	4.50	3.36	up
ASTEI20_032042	xanthine dehydrogenase-like	4648	3.35	6.99	up
ASTEI20_032249	UDP-glycosyltransferase UGT5-like, transcript	1944	22.68	20.87	up
ASTEI20_033905	cytochrome b-c1 complex subunit 8	556	2.88	3.52	up
ASTEI20_034193	UDP-glycosyltransferase UGT4-like	1991	12.31	11.07	up
ASTEI20_035158	venom carboxylesterase-6-like	1930	69.94	51.97	up
ASTEI20_035228	esterase E4-like	1958	5.54	6.30	up
ASTEI20_035931	chorion peroxidase-like	2536	−2.98	−3.23	down
ASTEI20_036049	venom carboxylesterase-6-like	2722	−6.20	−5.81	down
ASTEI20_039992	multidrug resistance-associated protein 1-like	5024	11.68	5.64	up
ASTEI20_040345	glutathione S-transferase 1-like	1773	10.54	13.14	up
ASTEI20_041459	probable cytochrome P450 6a14	1767	4.09	3.57	up
ASTEI20_041713	probable cytochrome P450 6a14, transcript	4297	5.75	8.04	up
ASTEI20_042864	protein arginine N-methyltransferase 5	2130	−3.35	−7.23	down
ASTEI20_044419	UDP-glycosyltransferase UGT5-like, transcript	2008	5.81	3.63	up
ASTEI20_044800	esterase B1-like	2161	−6.41	−13.64	down
ASTEI20_044864	glutathione S-transferase 1-like, transcript	1973	8.25	5.16	up
ASTEI20_046113	multidrug resistance-associated protein 1-like	4601	3.51	3.66	up

Note: R, resistant population (AWR); U, field-unexposed population (AWK); S, susceptible population (STE2); FC, fold change.

## Data Availability

The data supporting the findings of this study are available within the paper and its Supplementary Materials file. The original contributions presented in the study are publicly accessible. The RNA-seq data generated in this study have been deposited in the NCBI repository under accession number PRJNA1182831. BioSample accession numbers: SAMN44614196–SAMN44614201.

## Supplementary Materials

The following supporting information can be downloaded at https://www.mdpi.com/article/10.3390/genes15121603/s1: Figure S1: The mortality rates after insecticide exposure in the *Anopheles stephensi* field population in Awash Subac Kilo, Ethiopia; Figure S2: Violin plot of gene expression patterns for each population; Figure S3: Heatmap and hierarchical clustering of the 885 DEGs across the three groups; Figure S4: Venn diagram showing the 885 differentially expressed genes identified in the field *An. stephensi* population compared to the lab strain (STE2). AWK, field-unexposed; AWR, field-resistant; Figure S5: The determined threshold values for the construction of a biological correlation network. (A) Scale independence; (B) mean connectivity; Figure S6: Heatmap showing the five gene co-expression modules detected from the differentially expressed genes between the field-resistant population and the susceptible lab strain; Table S1: List of the 10,568 protein-coding genes expressed at a minimum exon read count of 5 across the 6 pooled samples; Table S2 List of the 885 significantly differentially expressed genes (DEGs) predicted from the comparison; Table S3: List of the 27 significantly enriched GO terms of the differentially expressed genes; Table S4 Gene co-expression network identified in the green module (weight > 0.40, 48 genes); Table S5 Gene co-expression network identified in the blue module (weight > 0.68, 48 genes).
