# Supplementary material for: Esterase-Mediated Pyrethroid Resistance in Populations of an Invasive Malaria Vector Anopheles stephensi from Ethiopia
Source: Genes (Basel). 2024 Dec 15;15(12):1603. doi: 10.3390/genes15121603 (PMC11675767; doi:10.3390/genes15121603)
Supplement: Supplementary file 1 [file genes-15-01603-s001.zip › genes-3368462_Supplementary material_Figure S1-S6.pdf]

Supplementary material

Figure S1 The mortality rates after insecticide exposure in the *Anopheles stephensi* field population in Awash Subac Kilo, Ethiopia. Data are represented as mean ± Standard deviation (SD).

Figure S2 Violin plot of gene expression patterns for each population.

Figure S3 Heatmap and hierarchical clustering of the 885 DEGs across the three groups.

Figure S4 Venn diagram shows the 885 differentially expressed genes identified in the field *An. stephensi* population compared to lab strain (STE2). AWK, field-unexposed; AWR, field-resistant.

Figure S5 Determine the threshold value for construction of a biological correlation network. A, Scale independence; B, Mean connectivity.

Figure S6 Heatmap showing the five gene co-expression modules detected from the differentially expressed genes between resistant field population and susceptible lab strain.

Table S1 List of the 10,568 protein-coding genes expressed at a minimum exon read count of five across the six pooled samples.

Table S2 List of the 885 significantly differentially expressed genes (DEGs) predicted from the two comparison.

Table S3 List of the 27 significantly enriched GO terms of the differentially expressed genes.

Table S4 Gene co-expression network identified in the green module (weight > 0.40, 48 genes).

Table S5 Gene co-expression network identified in the blue module (weight > 0.68, 48 genes).

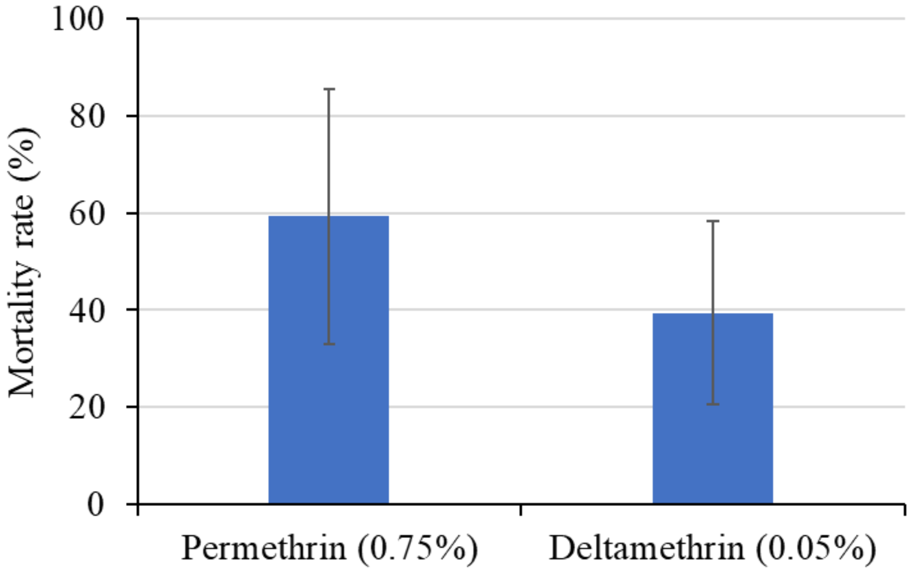

Figure S1. The mortality rates after insecticide exposure in the *Anopheles stephensi* field population in Awash Subac Kilo, Ethiopia. Data are represented as mean ± Standard deviation (SD).

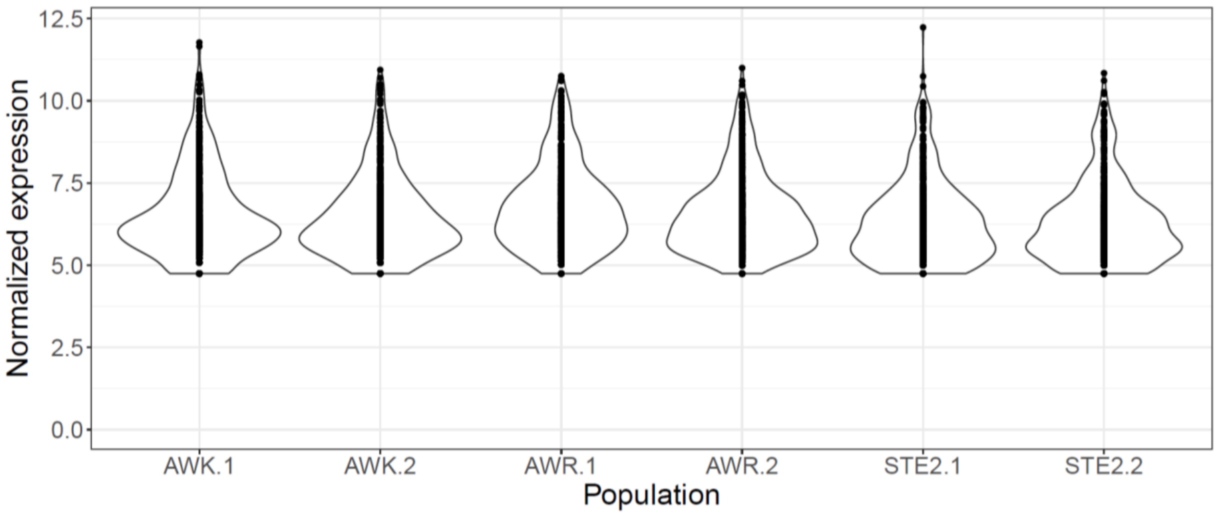

Figure S2. Violin plot of gene expression patterns for each population

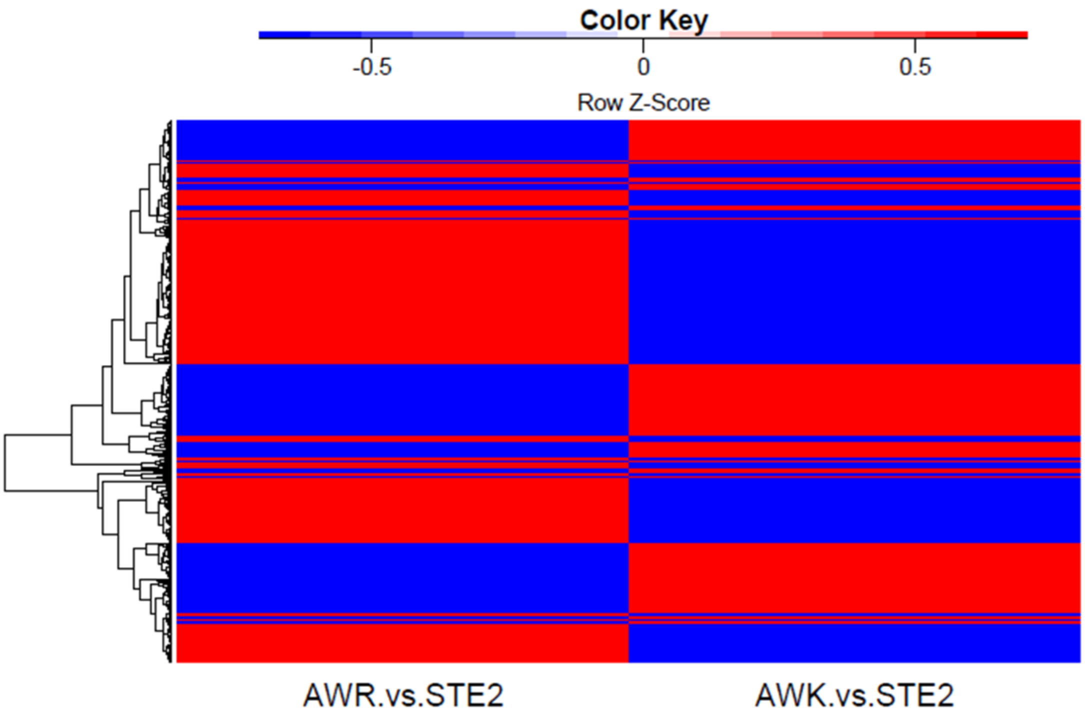

Figure S3. Heatmap and hierarchical clustering of the 885 DEGs across the three groups.

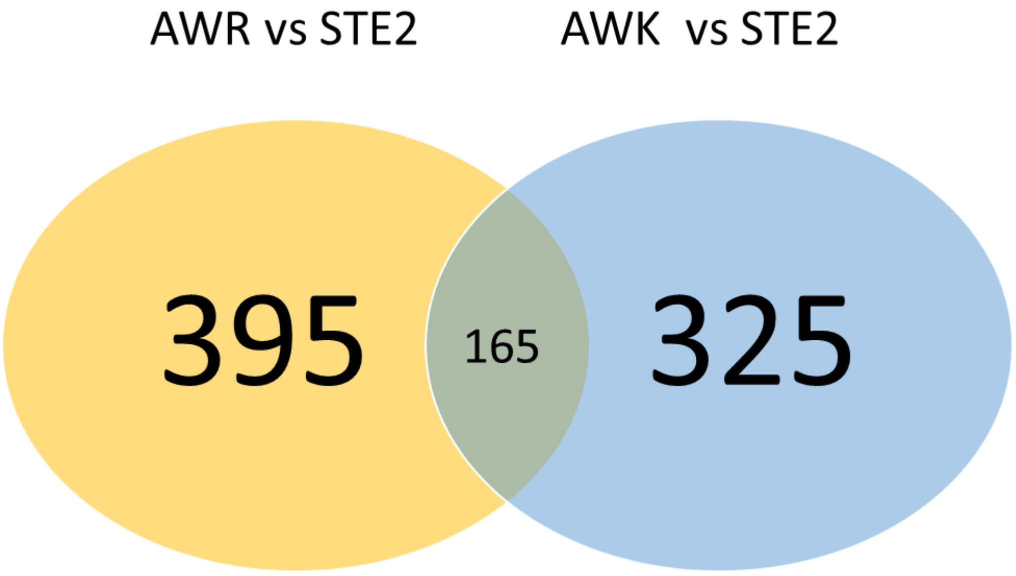

Figure S4. Venn diagram shows the 885 differentially expressed genes identified in the field *An. stephensi* population compared to lab strain (STE2). AWK, field-unexposed; AWR, field-resistant.

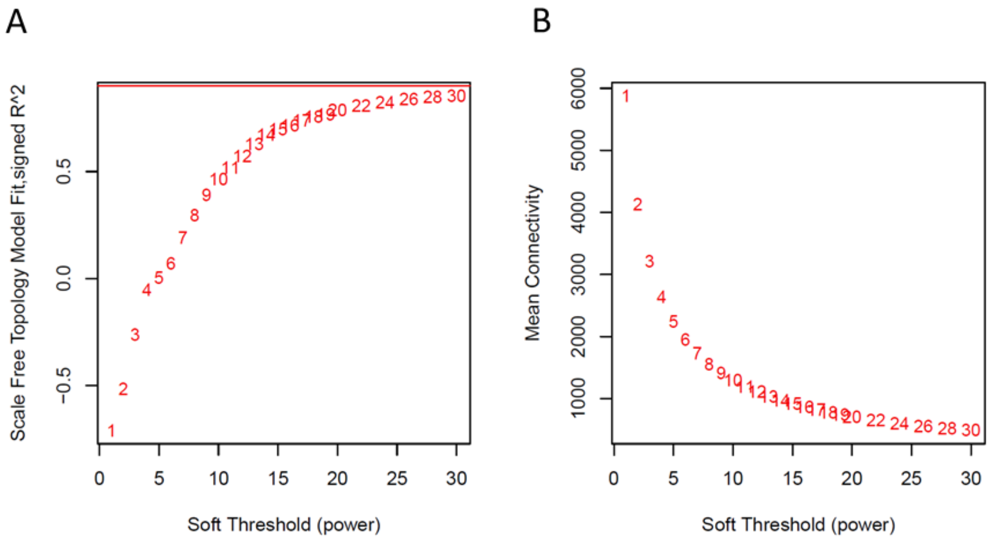

Figure S5. Determine the threshold value for construction of a biological correlation network. A, Scale independence; B, Mean connectivity.

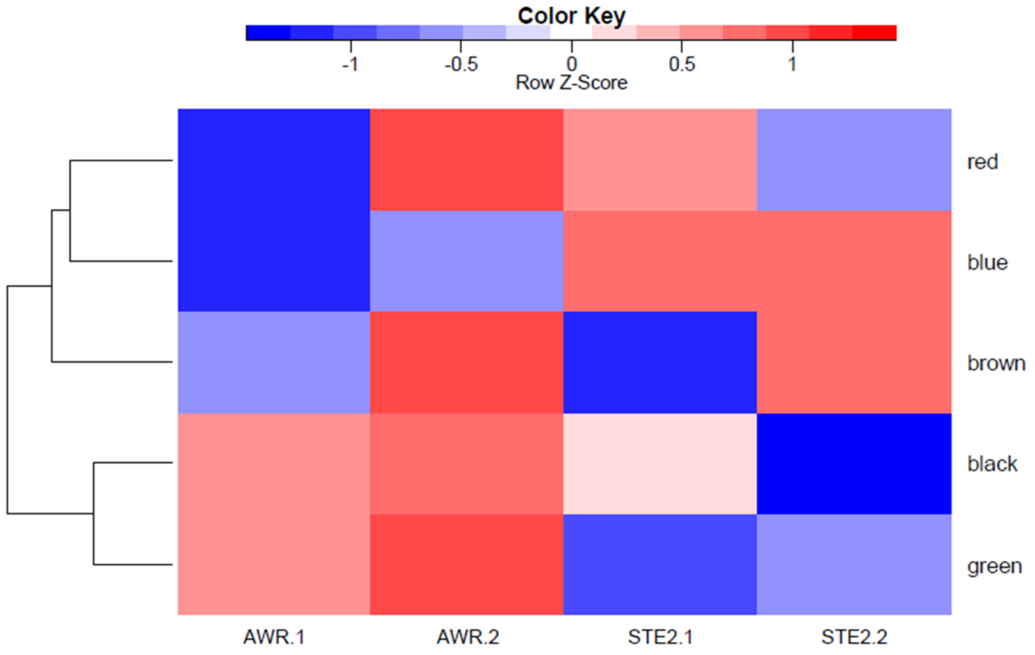

Figure S6. Heatmap showing the five gene co-expression modules detected from the differentially expressed genes between field-resistant population and susceptible lab strain.
